# Empowering the digital therapeutic relationship: virtual clinics for digital health interventions

**DOI:** 10.1038/s41746-018-0028-2

**Published:** 2018-05-16

**Authors:** John Torous, Honor Hsin

**Affiliations:** 1 Departments of Psychiatry and Clinical Informatics, Beth Israel Deaconess Medical Center, Harvard Medical School, Boston, MA USA; 2 Clinical Health Platforms, Verily Life Sciences, South San Francisco, CA USA

**Keywords:** Disability, Health occupations

## Abstract

As “digital phenotyping” and monitoring technologies begin to unleash the potential of data insights for mental health care, we propose here a complementary concept of the “digital therapeutic relationship” to unleash the power of the patient-provider alliance in clinical care. In millions of clinics today, care decisions are made on a daily basis in the context of a relationship honed through professional training to be respectful, protective, and empowering of patients. Now as clinical care evolves toward online and especially mobile platforms, it is critical to not ignore the digital therapeutic relationship and instead to realize that supporting it will require new and innovative means of care delivery. Here, we propose that technology can be harnessed to facilitate, augment, and expand these relationships directly, and identify virtual clinics as the currently missing but necessary environment to unleash the true potential of digital medicine.

## Introduction

On paper, digital health tools like smartphone apps appear to be the ideal solution to the current mental health crisis. Depression is now the leading cause of disability worldwide, and mental health disorders impact one in four people globally, yet current and projected future access to care and treatment remain inadequate. Today’s limited access to clinical care stands in contrast to the ubiquity of smartphones, heterogeneity in care delivery in contrast to standardized protocols of apps, and snapshot diagnostic assessments in contrast to continuous longitudinal monitoring by phone sensors. Digital phenotyping, the moment-by-moment quantification of individual-level human behavior and physiology in situ using data from smartphones and other personal digital devices^1^ offers a new window into understanding and addressing the real-world lived experience of illness. Yet the reality of digital health services like apps is different—uptake is low in clinics and engagement remains poor among the public.^2,3^ Searching for the cause of this striking disparity has proven difficult because it is neither tangible nor a fault in apps, clinicians, or patients. Rather we propose that a failure to address the digital interaction between patients and clinicians—the digital therapeutic relationship—and resulting lack of support for this new relationship limits the true potential of digital care.

## The digital therapeutic relationship

The digital therapeutic relationship for mobile health has been ignored because it is often invisible to those building apps. App developers create impressive apps that are marketed to clinicians or patients as a discrete tool. For example, most symptom monitoring apps log information but few facilitate the meaningful sharing of that information or are able to connect it to the electronic medical record. In small print in the terms and conditions of most apps, these products are careful to note they are not therapeutic tools, and that in case of an emergency the user should seek actual care with a health professional. Or scientists, often also with limited current patient contact, study apps in idealistic research settings that frequently ignore clinical realities. These apps, while impressive on paper, do not fare well when users are no longer paid for use, offered free phones, or given extra support as in studies. The result is a plethora of apps that are innovative, impressive, and even efficacious—but lack effectiveness or real-world applicability. The present climate of unprincipled machine learning, artificial intelligence, user centric design, or other features has not yet helped in bridging the gap.

Bridging the gap through addressing the digital therapeutic relationship is one pragmatic solution. Mental health clinicians respect the clinical utility of a strong therapeutic relationship and this relationship is a good predictor for treatment response across both medication and therapy interventions.^4^ While there isn’t a consensus definition of the therapeutic relationship, elements include mutual trust, alliance, respect, empathy, and positive regard between the patient and clinician.^5^ The current therapeutic relationship is developed in the brick and mortar clinic and strengthened through future in-person clinical visits. The nature of the clinical workflow, appointments, electronic medical records, liability, and billing reinforce the predominance of face to face therapeutic relationships. The clinical impact of therapeutic relationships spans all healthcare from psychiatry to surgery and is well summarized in a 1988 paper by Suchman and Matthews stating, “the connectional experience is basic to medical care”.^6^ This impact has also been quantitatively^7^ studied and often reported as a moderate effect across a range of conditions.^8^ The current model of apps as independent tools focused on either patients or clinicians,^9^ however, ignores the therapeutic power of this relationship, the reluctance of both patients and clinicians to abandon it, and the potential damage that can be caused to the relationship through fragmenting care.

The existence of digital therapeutic relationships is itself not new and supported by an abundance of high quality evidence from psychology, the social sciences, user experience, user interface, and design research.^10–12^ Perhaps it is no wonder that telehealth, an innovation that enables provider-patient contact remotely, is one of the most enduring technological advances today.^13^ But as smartphone apps and remote sensing technologies for health continue to rapidly expand, it is now time to revisit the digital therapeutic relationship in the context of mobile health.

## Supporting the digital therapeutic relationship

By reframing apps as tools to strengthen and augment the therapeutic relationship, it is possible to realize the full potential of these apps. Instead of creating apps that seek to duplicate current clinical services without offering any relationship (e.g., many cognitive behavioral therapy apps and symptom trackers), a new generation of apps that strengthen and inform the therapeutic relationship offers a host of novel uses. For example, reducing polypharmacy and in some cases stopping psychiatric medications, especially antipsychotic medications, is an important treatment goal^14^ that offers benefits of reduced medication burden and healthcare cost. But clinicians are justly cautious with such medication reductions and the mantra ‘the dose that gets you well keeps you well’ is frequently practiced in clinical care. At the same time, clinical wisdom informs that knowing a patient well and having a strong therapeutic relationship makes medication reductions less risky. Using an app to strengthen the therapeutic relationship in this case by offering both parties the ability to augment their face to face time with real time monitoring of symptoms, ability for immediate communication, and automatically triggered contingency planning could help clinicians and patients safely and confidently navigate medication reduction. The information the app would offer in this case matches shared therapeutic goals of patients and providers and facilities best practices instead of trying to work around them.

Like any relationship, the therapeutic relationship requires support and comes with responsibility. The clinician and patient must first develop a relationship based on trust. The most effective method for this remains an initial face to face visit. The digital therapeutic relationship still requires a responsible clinician which means that as in face to face care, the provider must assume clinical risk. Unlike today’s apps which disclaim any liability for adverse events or harm—the clinician is delivering medical and psychiatric care which means that he or she continues to assume the concomitant legal liability. This includes having a safety net for emergencies instead of the common disclaimer on today’s apps. It makes sense that the environment to support and foster the digital therapeutic relationship is not 100% virtual but rather a new hybrid that intertwines face to face and digital care. The virtual psychiatry clinic would see patients in person for new intakes but then prescribe apps that are monitored by clinicians. For example, response to and monitoring of medications would be done with apps with preset triggers to schedule an appointment or video visit if a lack of response is detected. Important lifestyle interventions like exercise that are critical to health could be remotely monitored and supported. Follow up visits would be primarily via tele psychiatry, but when necessary digital monitoring could trigger in person assessments. While remote and digital care would be encouraged, in person care would never be withheld and is necessary to some degree in order to maintain a strong relationship.

The purpose of the virtual clinic is to offer an environment conducive to digital psychiatry. Instead of forcing traditional clinics to adopt apps and other technologies^15^ in a setting that is not well suited to foster a digital therapeutic relationship, these new clinics would be established for exactly that purpose. Because the clinicians and workflow of the virtual clinic would be built around digital care and patients self-selecting for this type of care, an opportunity exists for the true potential of digital psychiatry to be realized. The goal of these clinics would be to realize the scalability of digital health tools like apps, deliver on the promise of increased access to care, and yet still offer effective clinical services that improve health outcomes. Thus, the success of these clinics would be determined by the ability to realize that potential in more efficient, higher quality, and reduced costs of care.

Significant challenges remain for this proposed solution, however. Scalability of the investment in this new model of care and integration with the existing landscape of resources (for example, inpatient hospitals or local psychosocial resources) are potential areas where a virtual clinic may fail in comparison to traditional healthcare settings. Ensuring consistency of care, effective multidisciplinary team structure, and continued adherence to standards of evidence-based practice will be crucial to any such endeavor.^16^ Last, whether an iterative process of digital creation can flourish in an environment where care delivery, not product development, is prioritized, remains to be explored.

## Conclusion

Virtual clinics that can support the digital therapeutic relationship offer an unexplored avenue to potentially realize the value of new technologies like smartphone apps for mental health as well as other conditions. This represents a shift in thinking of apps as tools that can themselves revolutionize care to viewing them as resources to facilitate and augment clinical relationships. On paper, digital health is easy—but the true test for these devices is not on paper but rather in delivering effective, efficient, and patient-centered care.^17^
